# Differentiation of the Follicular Neoplasm on the Gray-Scale US by Image Selection Subsampling along with the Marginal Outline Using Convolutional Neural Network

**DOI:** 10.1155/2017/3098293

**Published:** 2017-12-19

**Authors:** Jeong-Kweon Seo, Young Jae Kim, Kwang Gi Kim, Ilah Shin, Jung Hee Shin, Jin Young Kwak

**Affiliations:** ^1^Department of Biomedical Engineering, College of Medicine, Gachon University, Gyeonggi-do, Republic of Korea; ^2^Department of Radiology, Severance Hospital, Research Institute of Radiological Science, Yonsei University, College of Medicine, Seoul, Republic of Korea; ^3^Department of Radiology and Center for Imaging Science, Samsung Medical Center, Sungkyunkwan University School of Medicine, Seoul, Republic of Korea

## Abstract

We conducted differentiations between thyroid follicular adenoma and carcinoma for 8-bit bitmap ultrasonography (US) images utilizing a deep-learning approach. For the data sets, we gathered small-boxed selected images adjacent to the marginal outline of nodules and applied a convolutional neural network (CNN) to have differentiation, based on a statistical aggregation, that is, a decision by majority. From the implementation of the method, introducing a newly devised, scalable, parameterized normalization treatment, we observed meaningful aspects in various experiments, collecting evidence regarding the existence of features retained on the margin of thyroid nodules, such as 89.51% of the overall differentiation accuracy for the test data, with 93.19% of accuracy for benign adenoma and 71.05% for carcinoma, from 230 benign adenoma and 77 carcinoma US images, where we used only 39 benign adenomas and 39 carcinomas to train the CNN model, and, with these extremely small training data sets and their model, we tested 191 benign adenomas and 38 carcinomas. We present numerical results including area under receiver operating characteristic (AUROC).

## 1. Introduction

Thyroid cancer has been one of the most diagnosed forms of cancers worldwide over the past few decades [1]. Follicular thyroid cancer is the second most common thyroid cancer after papillary thyroid cancer, comprising 10–20% of thyroid cancer. It is noted that follicular thyroid cancer has a higher incidence of distant metastasis and thus has prognosis worse than the more common papillary thyroid carcinoma [2–4]. Therefore, it is important to preoperatively notice this entity for prompt management.

Follicular neoplasm of the thyroid gland comprises follicular adenoma and carcinoma. It is challenging to preoperatively differentiate these two entities, and much clinical effort has been made up to this point. Overlapping clinical presentations, ultrasound (US) features, and molecular biology resulted in a limited value of diagnostic power through preoperative evaluation with US, fine-needle aspiration cytology, and immunohistochemistry [5–8]. Therefore, a differential diagnosis of these two entities is currently obtained by identifying capsular or vascular invasion at the periphery of the lesion among pathologic examination following diagnostic thyroidectomy [9].

In CAD (computer-aided diagnosis), many scientists and researchers have developed methods to detect thyroid nodules or automated diagnosis assistance systems, mainly to differentiate between benignancy and malignancy of thyroid nodules and break through those difficulties in definitive diagnoses of nodule lesions and assist radiologists with developing a plan of action [10–12].

Recently, the rapidly progressing industries in artificial intelligence technologies reached numerous markets and countries in various fields of our life, even in the area of medical sciences [13–16]. In this article, we develop and demonstrate newly conducted techniques and observe some meaningful aspects seen in various experiments, such as scaling a parameterized normalization to draw reasonable evidence of the existence of features retained on the margin of thyroid follicular neoplasms, which could be helpful in identifying capsular or vascular invasion occurring at the margin of the lesion, or inspirational to the invention of an efficient numerical method to differentiate malignant from benign follicular neoplasms on US images, in view of a CNN (convolutional neural network) [17].

In this paper, after reviewing other machine-learning type methodologies in Section 2, we introduce our model training schemes, presented in Section 3, focused on a technique that disregards features of intro area of thyroid nodule images; that is, we concentrate our image recognition model on capturing the features characterized in the boundary region of thyroid follicular neoplasms, in virtue of the fact that the previously mentioned differential diagnosis based on the pathologic examination taken after diagnostic thyroidectomy depended considerably on the properties of the boundary region of the nodules. In Section 4, we present numerical results, developing a newly devised parameterized normalization treatment, including AUROC (area under receiver operating characteristic) and those curves, as well as overall differentiation accuracy, and so on. In Section 5, finally, we discuss the existence of features on the boundary of US thyroid follicular neoplasms that could possibly be trained by our proposed CNN based inference model and its efficiency, including our future works.

## 2. Technical Issues in US Classification Experiments Using Artificial Neural Network

In view of machine learning or artificial intelligent techniques for differentiation of malignant from benign thyroid nodules, there are lots of methods or treatments with sample data sets to extract efficient features for application in a training model of a given machine learning or ANN training tools [10, 11, 18–20]. For support vector machine (SVM), some remarkable ways of feature extracting techniques and imagery subsampling treatments are conducted to efficiently train classification models such as those found in [10, 20–23], and, for ANN type of methods, the methodologies found in [10, 19, 24–27] mostly use some ways of preprocessed training with feature extraction techniques including pathological reports or information on patients such as age, sex, health condition, and the results of various medical tests or cytological data. In other words, most of ANN methods found in there actually demonstrate training with nondirect US images but with some kinds of nonimagery input data sets extracted from original US image information.

**Figure 1:**
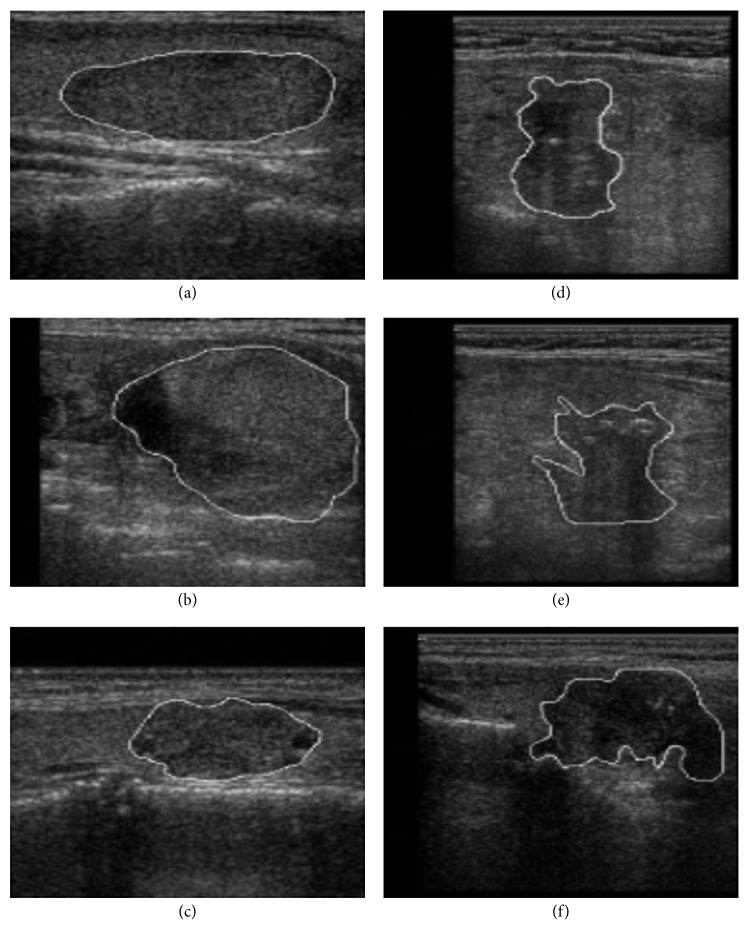
Thyroid US images with delineated nodules: (a–c) nodules of regular boundaries; (d–f) nodules of irregular boundaries, belonging to the data set in [21].

In our implementation of CNN model training for differentiating between thyroid follicular adenoma and carcinoma for US thyroid images, we engage US images in a fixed size of pixels in resolution on input nodes directly without extracting any preprocessed statistical features. For a training object of a CNN model, from the reported diagnostic US determining features in the differentiation of thyroid follicular adenoma and carcinoma, we focus on a way of training which magnifies training efficiency of imagery and morphologic features of US found in the adjacent region of the boundary of lesion. For a method of SVM applied in [21] to differentiate risky hypoechoic thyroid nodules, although they try to take the features found in boundary region of thyroid nodules by setting up the data set comprising 131 medium-risk hypoechoic nodules characterized by regular boundaries and 42 high-risk hypoechoic nodules characterized by irregular boundaries, since the morphological shapes of boundary regions are so distinctive that even human eyes may easily recognize the risky nodules, one may not be sure that its model would be a good fit to work for any ambiguously shaped general cases of thyroid follicular adenoma and carcinoma (refer to Figure 1).

Exhibited here are renderings of our own sample gatherings of thyroid nodule images to deal with our classification models of convolutional neural network, and, afterward, we introduce and define the type of training methodology in Section 2.

For our own collection of sample thyroid images, we have 250 cases of follicular adenoma, as well as 83 cases of follicular carcinoma, visualized in gray-scale 8-bit bitmap US thyroid nodule images, and the data sets were obtained from 2 different US clinics which identified as Hospital A (= H_A_) and Hospital B (= H_B_) (refer to Table 1). For the data denoted by clinic HA, in total, 230 patients with 230 thyroid nodules were included in this study. Of the 230 patients, 51 (22.174%) were men, and 179 (77.826%) were women. Mean age of the 230 patients included was 48.72 years. Mean size of the 230 thyroid nodules was 29.84 mm, and the mean of the pixel intensity of the grey-scale 8-bit bitmap US images is 63.819, where the mean value of the max intensity is 176.1475, and the mean of the minimum intensity is 7.1230. For the data of HB, totally, 103 patients with 103 thyroid nodules were included in this study, where 22 (21.359%) were men, 71 (68.933%) were women, and 10(9.708%) were the missed sex identification, and the mean age was 43.90 years. Mean size of the 103 thyroid nodules was 32.81 mm, and the mean of the pixel intensity of the grey-scale 8-bit bitmap US images is 82.07 where the mean value of the max intensity is 192.1154, and the mean of the minimum intensity is 6.6827. These data sets are given from both institutional databases which was reviewed after from January 2003, for patients diagnosed with follicular adenoma and follicular carcinoma after surgical excision. In Table 1, we present the list of the numbers of our sample cases of US thyroid images.

## 3. US Differentiation Applying CNN

We make use of CNN to differentiate US images of follicular neoplasms between the adenoma and the carcinoma. We demonstrate experiments with the data set given in Table 1 to train a CNN model to infer the differentiation.

### 3.1. Data Setup

#### 3.1.1. Making Subsets

Here, aiming to derive a data invariant numerical result related to the characteristics of the fine imagery features captured by our CNN model retained on the margin of thyroid follicular neoplasms, delivered from various examinations as far as possible, we organize 6 kinds of disjoint subsets from the data set given in Table 1, into Set_*a*, Set_*b*, Set_*c*, Set_*d*, Set_*e*, and Set_*f* (see Table 2).

After removing some US contaminated images tainted at some marginal area with an extraneous substance, such as diagnostic marking signs of the radiologist, we reduced the data sets shown in Table 2 into those refined sets listed in Table 3, in which Set_*a*^*∗*^ corresponds to Set_*a*, and Set_*b* to Set_*b*^*∗*^, and so on.

#### 3.1.2. Training Data and Test Data

**Figure 2:**
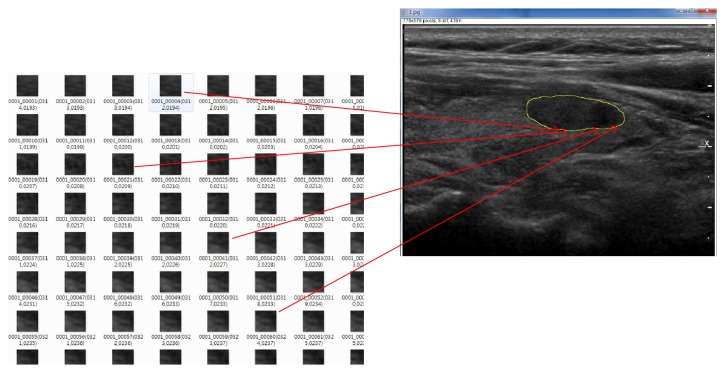
Selection of images (here we set 50 × 50 pixels in size) aligned on the contour of each thyroid follicular neoplasm's margin.

To implement the training of our model, we use Set_*a*^*∗*^ as training data and the other subsets for each as test data, based on the data sets given in Table 3; that is, this organization of training and test data is set to be an extremely small training set for small test set architecture to demonstrate various examinations and to deduce the existence of data invariant characteristics of fine common features captured by our nodule's boundary based CNN modeling. To set up the practical training and test data sets based on each boundary of nodule, we select small 2D box images (here we set 50 × 50 pixels in size) aligned on the contour of each thyroid follicular neoplasms' margin (see Figure 2).

To have this selection of marginal box images for the training data, following the contour of the nodule's margin, we chose somewhat distinctive images judged manually, while for test data we select box images centered at every point of pixels on the manually drawn, closed virtual contour margin line of the thyroid nodule, and afterward we have the training and test data sets given in Table 4, in which Set_*a*° corresponds to Set_*a*^*∗*^, and Set_*b*° to Set_*b*^*∗*^, and so on.

### 3.2. Differentiation via the Rule of Decision by Majority

**Figure 3:**
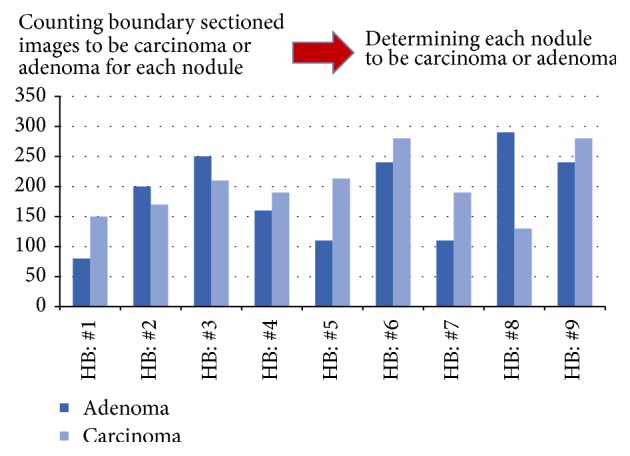
An illustration to determine differentiation of nodules by counting CNN model based semijudged selection images taken from boundary regions for each nodule.

From the nodule information given in Table 3 and the training and test data organization given in Table 4, we examine the differentiation, applying a decision by majority to judge the differentiation for each follicular neoplasm by those subsampled data sets taken from each own boundary region. For a simple representation of our CNN based statistical inference applying the decision by majority, let us assume that there exist 500 selected subsampled images given from the boundary of a nodule so that our trained CNN model determines each selected subsampled image to be carcinoma in 255 counts and adenoma in 245 counts, and then we determine that the nodule is carcinoma, owing to the fact that the counts to be carcinoma exceed those for adenoma (see Figure 3).

#### 3.2.1. The Structure of Convolutional Neural Network as a CNN Model

**Figure 4:**
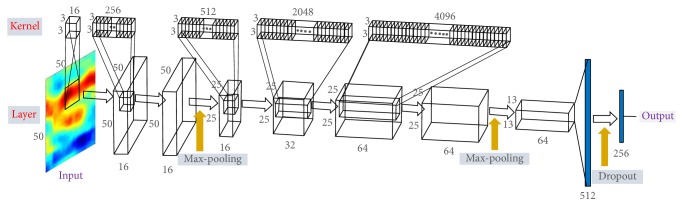
CNN training architecture with 5-conv, 2-pool, and 2-fully-conn. network corresponding to the structure in Table 5.

We apply an AlexNet type of CNN structure [28] to train data sets, which comprises 5 convolutional layers and 2 pooling layers, the details of which are described in Table 5 and Figure 4. (In Table 5, characters *m* and *n* represent the size of the convolution kernel for each input channel and the number of total kernels applied to each layer, resp.)

### 3.3. Overview

In view of the setup, the data set is organized from an assumption that every margin of thyroid follicular neoplasms may contain certain obvious features that help differentiate between adenoma and carcinoma and that those features would well be detected and trained, even with the small number of images of thyroid nodules [9]. Our standard of outlining of the contour of each thyroid follicular is drawn from the official medical specialist from both clinic, Samsung Medical Centre, and Yonsei University Medical Centre in Seoul, South Korea, the coauthors of this article.

## 4. Numerical Results

In this section, we present numerical results related to differentiating thyroid follicular neoplasms between adenoma and carcinoma and some observable aspects in the feature recognition of CNN in view of a newly developed data normalization method by devising a parameterized scaling treatment. For the numerical results in this section, we train the CNN model described in Table 5 and Figure 4, with 380 of epochs of training, 400 of batch size, 0.0001 for learning rate, and 0.5 for dropout rate, with a standard backpropagation algorithm [17, 28, 29]. We customized the popular TensorFlow (version 1.0.0) library in Python3.x for our main programs of the experiments. It took several minutes to train each experimental model where it took a few seconds to infer the results for test data sets, on two Ndvia Pacal TitanX 12 GB GPUs.

### 4.1. Training Aspects of the Parameterized Scaling Treatment in Data Normalization

Here, we give training results of CNN with regard to the data normalization, applying a parameterized scaling treatment. For the normalization of training data in our experiments, we apply a mean-zero based min-max normalization of training input data, which transforms all the scores of input data into a common range [0, 1] and then minus the mean of the input data set. We let a pair of indices (*i*, *j*) represent the pixel point located in the ith position in the *x*-axis and the *j*-th position in the *y*-axis in each input image and the corresponding pixel value is denoted by *u*_*ij*_; then the mean-zero based min-max normalization *v*_*ij*_ for training data is given as

(1) $$\begin{array}{c} v_{ij}=\frac{u_{ij}-E\left[u_{ij}\right]-{min}_{\left(i,j\right)}u_{ij}}{{max}_{\left(i,j\right)}u_{ij}-{min}_{\left(i,j\right)}u_{ij}}, \end{array}$$

where *E*[*u*_*ij*_] denotes the mean value of *u*_*ij*_ in the position (*i*, *j*).

While the test data is normalized applying a scaling parameter *α*, it is performed as

(2) $$\begin{array}{c} q_{ij}=\frac{p_{ij}+\alpha \cdot E\left[p_{ij}\right]-{min}_{\left(i,j\right)}p_{ij}}{{max}_{\left(i,j\right)}p_{ij}-{min}_{\left(i,j\right)}p_{ij}}, \end{array}$$

where *E*[*p*_*ij*_] denotes the mean value of *p*_*ij*_, the pixel value of test data is at position (*i*, *j*), and *q*_*ij*_ denotes the parameterized normalization of *p*_*ij*_. Here, note that if *α* = 0 in (2), it is the min-max normalization [30].

**Figure 5:**
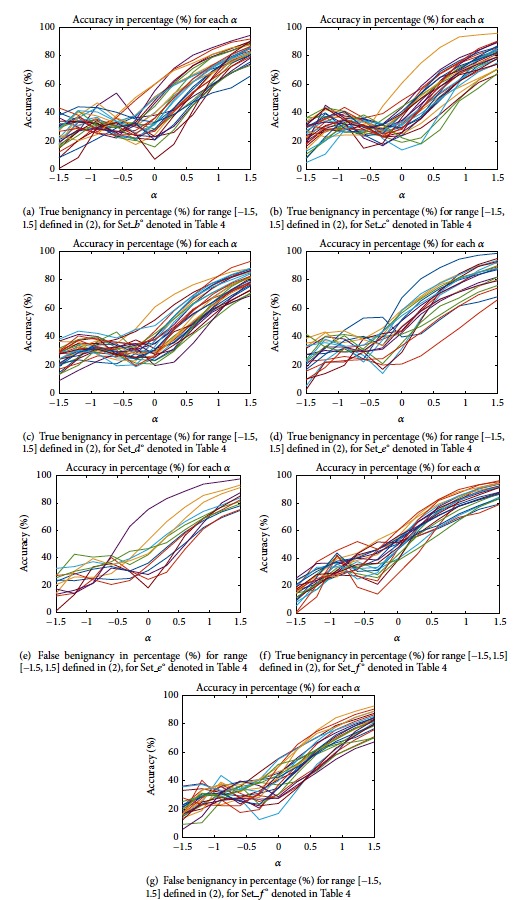
Plots of differentiation in percentage (%) versus *α* for false benignancy of carcinoma and true benignancy of adenoma for each of the test data sets.

Here we are examining the CNN model for the test data. We have the parameter *α* in (2) range [−1.5, 1.5] for every 0.3 increase. For the results obtained by test data from Set_*b*° to Set_*f*° listed in Table 4, we present the accuracy of differentiation in percentage (%), and for each test set we draw the plots given from Figures 5(a)–5(g), where we draw plots of true benignancy of adenoma for Set_*b*°, Set_*c*°, Set_*d*°, Set_*e*°, and Set_*f*° and the false benignancy of carcinoma for Set_*e*°, and Set_*f*°, respectively. In Figure 5, each curve represents the tendency of differentiation for a corresponding single follicular nodule; for example, for Set_*b*°, there are 30 kinds of nodules (refer to Table 3), and then there are 30 lines of curve in Figure 5(a), and for a given *α* each plot lying in the vertical line indicates the percentage (%) to be classified as benign, one for each nodule, respectively.

**Figure 6:**
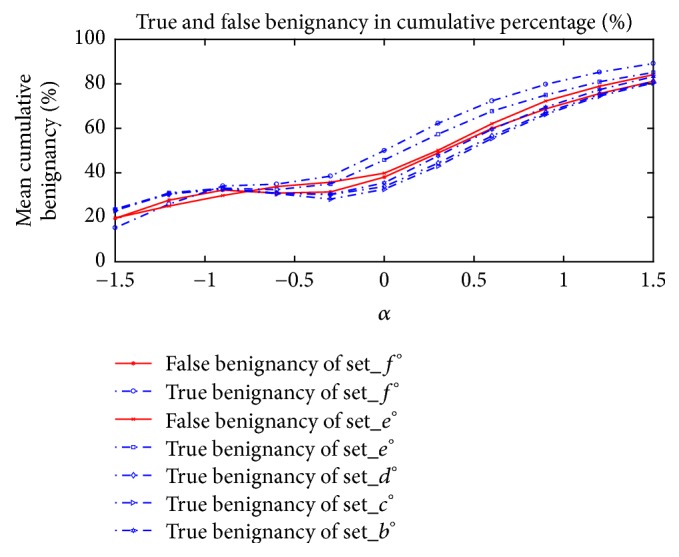
Plots of true benignancy of adenoma for Set_*b*°, Set_*c*°, Set_*d*°, Set_*e*°, and Set_*f*° and false benignancy of carcinoma for Set_*e*° and Set_*f*°, in cumulative percentage (%) for *α* ranging [−1.5, 1.5] defined in (2).

Now, summarizing the plots given in Figure 5, we draw the plots in mean cumulative percentage (%) versus *α* for true benignancy of adenoma test data and for false benignancy of carcinoma data, observing the slopes of plots in the mean cumulative percentage (%) proportional to *α*, which represents the tendency of differentiation to be classified as benign adenoma. We provide the plots to compare those slopes in Figure 6.

Seeing the plots in Figure 6, the slopes of mean cumulative percentage (%) versus *α*, where *α* ≥ −0.5, have a positive sign for all the plots, and these behaviors of slopes could promote the increase of differentiation accuracy in total for true benign data, but the behavior could also cause a decrease for carcinoma data, which gives us a sense of fine-tuning through the control of *α*.

### 4.2. Fine-Tuning Effect of the Parameterized Data Normalization

Along with the fact that the control of *α* could give an increase in total differentiation accuracy, the result of a demonstration of differentiation for a set of test data reveals the possibility that a nice choice of *α* gives us a highly recommendable CNN differentiation model as a model of fine-tuning. Here, a result of the demonstration conducted on test data Set_*f*° is given in Table 6, for which we choose *α* = 0.15.

**Figure 7:**
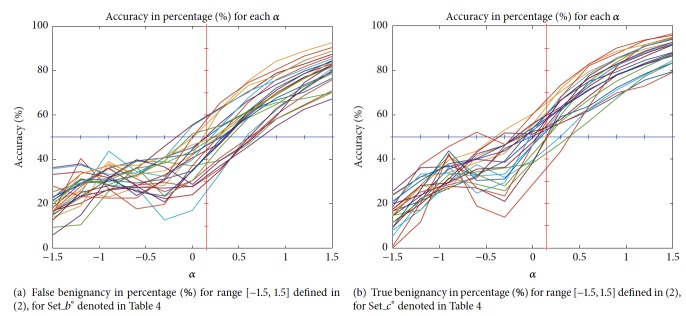
Plots of differentiation in percentage (%) versus *α* for false benignancy of carcinoma and true benignancy of adenoma for test data Set_*f*°.

In Figure 7, we give the plots of differentiation in percentage (%) versus *α* for false benignancy and true benignancy for test data Set_*f*°. Seeing Figure 7(a), we know that around *α* = 0.15 the plots lying in vertical line with values less than 50% counts about 19, and, seeing Figure 7(b), we know that around *α* = 0.15 the plots lying in vertical line with values greater than 50% count 17 approximately.

**Figure 8:**
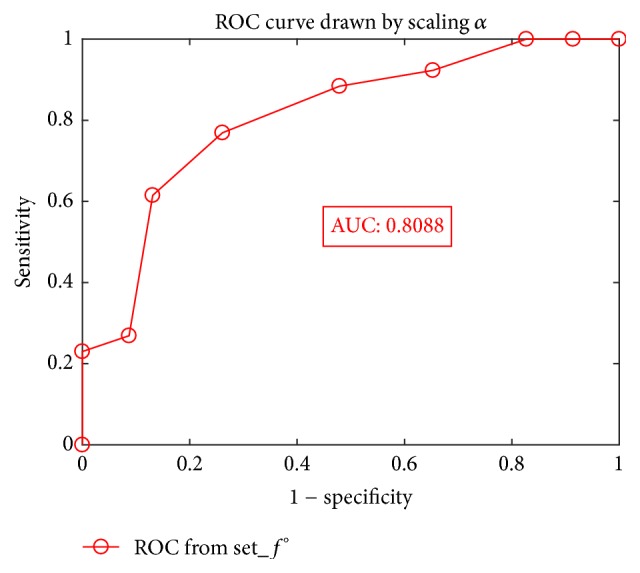
ROC curves given by differentiation test on Set_*f*°, for *α* ranging [−0.6, 0.6] defined in (2).

Furthermore, to represent the efficiency of our training model and the comparison result given from different values of *α*, in Figure 8, we give the receiver operating characteristic (ROC) [31] curve drawn by the differentiation result from the test on the test data set Set_*f*° by scaling *α* in the interval of [−0.6, 0.6], where the corresponding area under the curve (AUC) is 0.8088.

On the other hand, seeing that test data sets Set_*b*°, Set_*c*°, and Set_*d*° are derived from the data set H_A_ and Set_*e*° and Set_*f*° from H_B_, respectively, we apply a different normalizing parameter *α* in (2) for the sets from H_A_ and for those from HB such that *α* = 1.5 for H_A_ and *α* = 0.15 for H_B_. The differentiation results for both H_A_ and H_B_ are given in Table 7.

## 5. Discussion

In our experiments of CNN inference modeling to differentiate thyroid follicular neoplasms between follicular adenoma and carcinoma of gray-scale 8-bit bitmap US thyroid images, we implemented the mean-zero based min-max normalization method defined in (1) for input data to be trained by CNN architecture and rescaled it with a parameter denoted as *α* in (2) for test data. In our numerical simulation of training of model, referring to Table 3, the readers may see that our acquisition of the training data and test data sets is taken from two different clinic centres, the total amounts of samples for the use of training data set are very limited, the whole samples of follicular carcinoma images from clinic H_A_ are used to be training data, and the sample images from H_B_ are used to be test data set, so that we naturally determined the fixed partitioning scheme. As a result of the experiments of scaling the normalization parameter *α* chosen in a real number interval [−1.5, 1.5], we found out that the slopes of mean cumulative percentage (%) versus *α*, where *α* ≥ −0.5, have a positive sign for all the plots, and these behaviors of slopes increased the differentiation accuracy in total for true adenoma data but promoted a decrease for carcinoma data, providing a sense of fine-tuning through the control of *α*. Although the training data is chosen among the subsets of H_A_ by adjusting the normalizing parameter *α* chosen differently from each other between the two hospital data sets, H_A_ and H_B_, respectively, we could differentiate the images in H_B_, of which the test result of differentiation over 89% in overall accuracy supports the availability of our inference model. Furthermore, from the test results shown in Figure 6, we see that there is no pairing of data sets, of which plots have to cross over themselves where *α* ≥ 0, of which the original hospital databases are different from each other, and these plot behaviors in the results might somewhat weakly suggest that the two different hospital databases have their own distinctive imagery characteristics for each of them so that it makes sense to apply a different normalizing parameter *α* for each hospital data set, respectively. For this, one may suggest that the configuration of the pixel intensities which differs along both data sets, HA and HB, affects that. (Refer to the fact that, for HA, the mean of the pixel intensity of the grey-scale 8-bit bitmap US images is 63.819, the mean value of the max intensity is 176.1475, and the mean of the minimum intensity is 7.1230, whereas, for HB, the mean of the pixel intensity is 82.07, the mean value of the max intensity is 192.1154, and the mean of the minimum intensity is 6.6827, as denoted before.)

On the other hand, with regard to the data set, our shortage of data sets seldom makes someone imagine a good performance to infer disease diagnostic determination, comparing to that of such a relatively plentiful of data sets of MNIST and ILSVRC [32]. Hence, to tackle our small data set problem, we mainly seek to develop inference methodologies and overcome the extremely harsh task of our inference model with small data set via seeking a kind of ensemble-like neural-network method. Moreover, for the performance of our proposed model, basically like other machine learning based technology, we may not be sure about the robust functioning of our methodology yet, since like most of other vision based deep-learning architectures severely it suffers from the types of organizations or the amount of sample data sets to be applied to do specific inference, so that the proposed methodology may or may not suffer from those kinds of problems. In our research article, we have not suggested any mathematical proof of theoretical issues related to our presented numerical results rather than given experimental conviction for the possibility of the utility. From the experiments in [5], also we see that although the amounts of samples are so rare, they conclude some reasonable researching insights into the diagnostic differentiation for follicular neoplasm lesion of thyroid. Now we hope that we open the chances of the successful application similar to our proposed method to the readers with much plentiful sets of sample data.

For the sample data acquisition, both health centres, here Hospital A (= H_A_) and Hospital B (= H_B_), referring to Table 1, have different protocol for the acquisition of the ultrasound images, based on the apparatus to take the ultrasound image pictures; that is, the machines to take the ultrasound images and the related mechanical conditions are different. In this case, we have the difficulty to adjust the data sets to have the same depth of intensity of ultrasound wave and resolutions for both clinics' data sets, and we thought that the differences in those parameters influence the inference model results, and it is expressed in the classification results where the classification results for data sets included either side of clinic have the similar up-and-down slopes of differentiation, that is, for data from same clinic have the tendency of near distance of plots themselves relatively compared to the other clinic's data sets, referring to Figure 6.

For the sample data organization, referring to both clinics' data sets, the critical point to determine how many data sets to be set as training data and test data is largely dependent on the number of follicular carcinoma images, since, to balance the number of sample data for training the model, we set prior data from either clinic (here H_A_, referring to Table 3) having much ample number of samples compared to the other clinic (here H_B_, referring to Table 3) to be used as training data, without loss of generality. And the total amount of follicular carcinoma sample images are be used in developing our inference model inferior to that of follicular adenoma images so that we determine having training data set from the sample images of H_A_ which owns further sample data compared to H_B_, especially for follicular carcinoma images. Actually, considering the data confusion in training the inference model occurred from the mixed data given from different environment of protocol in data acquisition from the two different clinic centres and, to avoid that ill-conditioned data organization and the following training results, we mainly separated the training data set given from either clinic and the test data set from the other clinic. And lastly, we determined organizing the training data and the test data as given in Table 3.

**Figure 9:**
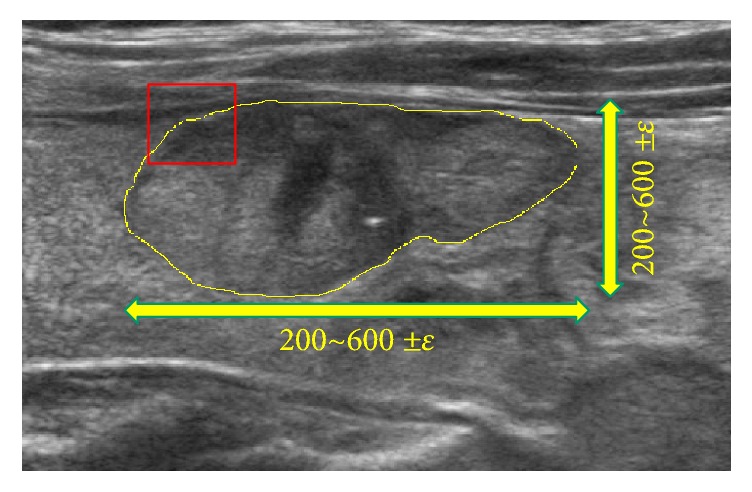
An example of a raw contour ROI of US thyroid image with resolution size ranging 200~600 ±  *ε* pixels. The red square represents an example of 2D box image we have selected to set up the data sets for the use in developing our deep-learning inference model, which is described in Section 3.1.

Now, here we give an overall answer to handle our choice of hyperparameters for our proposed neural network. Referring to Figures 5 and 6, we found out that the tendency of the slopes in those plots in Figures 5 and 6 gives us that as the proposed normalization parameter *α* moves the differentiation results change, and those kinds of differentiation trends are revealed to be coherent to each model with some variances of the neural network's parameters such as batch size and learning rate. Consequently, our proposed values of the neural network's parameters are one of the good choices which enabled us to get the numerical results which are persuasive to readers to convince them of the effectiveness of our proposed methodology to infer the differentiation depending on our organization of data sets. In our experiments, we experienced some overfittings or underfittings for the validation sets for training epochs over just several hundreds of epochs, and the similar phenomenon often happened for some variances of learning rates, and so on. For dropout rate, (the recently introduced technique, called “dropout” [29], consists of setting to zero the output of each hidden neuron with probability 0.5. The neurons which are “dropped out” in this way do not contribute to the forward pass and do not participate in backpropagation), we refer to the dropout rate given in [32] which deals with the AlexNet. For the structure of CNN, in our experiments, there is no prominent dominance for many heavy layers of CNN rather than popular AlexNet type of CNN architecture. For the 2D box image of size 50 × 50 pixels, as we see the illustration given in Figure 9, the raw contour ROI of US images taken from both clinic centres has the resolution size about 200~600 ±  *ε* pixels, and we thought that the resampling 2D box image, which is represented as the red square in Figure 9, (to be inferred for the full US image's differentiation based on our ensemble-like voting system of CNN) should be not too small or too large to have the inference model not to lose the critical morphological vision based features which may reside in the region of boundary of thyroid lesion. And of course, even our choice of the 2D-boxing size is not absolutely given someone to ensure it is the best choice, since the size may be the one of good choice to infer the model. Unfortunately, like most of other deep-learning models, especially for vision based models like CNN, there are still behaviors of each model's distinctive inference performances, and someone may say it is just black-box to analyze it in the sense of mathematical inspirations.

On the other hand, out of loss of generality, the choice of our neural network's parameters does not guarantee the absolute superiority for our applied AlexNet types of neural network; it is only dependent on one's own data sets and the experimental experiences and, here in our proposed method and the corresponding numerical results, only made to give the readers sorts of insight about the possibility or the effectiveness of our proposed inference model.

For the experimental experiences, we have ever applied various kinds of examinations with SVM, K-NN, simple ANN, and so on. Unfortunately, with these activities of experiments, we did not find any acknowledgeable results of inference models, yet. Finally, as we apply our proposed methodology, we observed breakthrough results, although still one may be doubtful of the real big data based performance of it. These results of our proposed method to infer the diagnoses to determine the alternative choice of classification problem, showing a possible superior task ability of ensemble-like methods to normal classical inference methodologies generally known.

### 5.1. Comparison with the Benchmark Thyroid Follicular Neoplasm US Images

#### 5.1.1. Preliminary Experiments by SVM, KNN, ANN, and CNN

As mentioned above, we have applied various kinds of basic examinations with SVM, KNN, Normal Bayes Classifier, and Feed-Forward-Perceptron network (ANN) to have similar types of differentiation of thyroid follicular neoplasm US images, based on the sense of full size image and not resampling from the contour region of nodules. The preliminary results of SVM, KNN, Normal Bayes Classifier, and ANN which applies with some well-known feature selection such as Mean, Skewness, Energy, Entropy, Compactness, Solidity, GLCM_contrast, GLCM_homogeneity, GLCM_energy, GLCM_entrophy, and Gabor_O2S1 are given in Table 8 [33, 34]. The readers may well compare the results to those in Table 7.

And even from the preliminary experiments taken with the full US image based (not resampled along contour) CNN inference, we have found the total accuracy ~75%, but there are still many follicular carcinoma images that failed to be differentiated.

#### 5.1.2. Comparison with USFNA Based Differentiation for a Follicular Thyroid Neoplasm US Images

For the comparison performance of our differentiation method for US images follicular thyroid neoplasm, we have found the USFNA (ultrasound-guided fine-needle aspiration) and the experimental results in [5] where the FNA performance ranges 51~67% in accuracy, which gives inferior results compared to our proposed methodology, as given in Table 9.

On the other hand, we found our general types of benchmark computer-aided systems listed in [35] where the author collected sample images from the open database proposed by Pedraza et. al. [36]. They applied a pretrained model transferring model which is initialized from the pretrained GoogLeNet network achieving excellent classification performance attaining 98.29% classification accuracy, 99.10% sensitivity, and 93.90% specificity. Although the types of US thyroid images of various computer-aided differentiation systems found in [21–23, 35] present excellent performances, their models are mostly treated with papillary thyroid carcinoma. And there are lots of reports that even USFNA is widely used in discriminating between benign and malignancy in various lesions of the thyroid showing excellent performances (sensitivity 65%–98% and specificity 72%–100%) for papillary thyroid carcinoma [5].

## 6. Conclusion

Although the amount of data sets relatively is not so plentiful compared to some well-known big data based machine-learning models, by the concurrent research works in the reference's authors where the follicular thyroid neoplasm US images are still not well studied for deep-learning based inference technology, we conclude that our proposed methods of CNN with data sets given by image selection subsampling along with the boundary of thyroid follicular neoplasms may detect some morphological features reflected in the region of boundary of nodules, which make sense to be supported by the background knowledge related to the known US image features indicating the criteria for diagnosing the carcinoma of thyroid follicular neoplasms in the general sense of clinical reports, especially concerning the characteristics of the marginal contour region of thyroid follicular neoplasms.

## 7. Future Works

Meanwhile, these results also reveal a suggestion that some imagery features, which could be recognized as scaling *α*, exist on the boundary of nodules so that a CNN inference model recognizes them and learns. These conjectures of the existence of learnable imagery features adjacent of the boundary of nodules for our CNN model need to be proven by a variety of fine-tuning techniques, including Standardization (*Z*-score normalization), tanh-Estimators, and other data normalizing techniques [37], as well as adjusting batch training modes, learning rate, convolution layers, and so on. Moreover, although we fixed the pixel resolution in this article to 50 × 50 for the subsampling image selection near the boundary of nodules, one may have other flexible choices of subsampling image size to train CNN and compare the efficiencies.

## Figures and Tables

**Table 1:** Configuration of the list of the numbers of our sample collection of ultrasonography thyroid nodule images without sex identification.

	Hospital A	Hospital B	Total
Follicular adenoma	190	60	250
Follicular carcinoma	40	43	83

**Table 2:** Configuration of the list of the numbers of our sample collection of US thyroid nodule images in 6 disjoint subsets.

	Set_*a*	Set_*b*	Set_*c*	Set_*d*	Set_*e*	Set_*f*	Total
Follicular adenoma****	H_A_				H_B_		250
	40	30	60	60	30	30	
Follicular carcinoma	H_A_				H_B_		83
	40	0	0	0	13	30	

**Table 3:** Refined configuration of the list of the numbers of our sample collection of US thyroid nodule images in 6 disjoint subsets.

	Set_*a*^*∗*^	Set_*b*^*∗*^	Set_*c*^*∗*^	Set_*d*^*∗*^	Set_*e*^*∗*^	Set_*f*^*∗*^	Total
Follicular adenoma****	H_A_				H_B_		230
	39	30	59	59	20	23	
Follicular carcinoma	H_A_				H_B_		77
	39	0	0	0	12	26	

**Table 4:** The number of selected partial box images along with the contour of margins of thyroid follicular neoplasms used to organize training and test data sets.

		Follicular adenoma	Follicular carcinoma	Total
Training_data	Set_*a*°	625	859	1484

Test_data	Set_*b*°	18170	0	18170
	Set_*c*°	43669	0	43669
	Set_*d*°	50061	0	50061
	Set_*e*°	12537	8939	21476
	Set_*f*°	18740	16648	35388

**Table 5:** Training structure of the convolutional neural net (5-conv, 2-pool, 2-fully-conn structure).

Layer	(*m* × *m*) × *n*	Activation	
Conv.	(3 × 3) × 16	ReLu	
Conv.	(3 × 3) × 256	ReLu	
Max-Pooling	kernel size: (2 × 2)		Strides: 2
Conv.	(3 × 3) × 512	ReLu	
Conv.	(3 × 3) × 2048	ReLu	
Conv.	(3 × 3) × 4096	ReLu	
Max-Pooling	kernel size: (2 × 2)		Strides: 2
Fully-Conn.	512	ReLu	
Fully-Conn.	256	ReLu	(Dropout rate: 50%)
Fully-Conn.		Softmax	Output units: 2

**Table 6:** Result of the CNN inference conducted on test data Set_*f*°, applying *α* = 0.15.

	Predicted_Adenoma	Predicted_Carcinoma	
Set_*f*°	*True_Adenoma* *17*	*False_Carcinoma* *6*	Accuracy (True negative rate) 73.91%
	*False_Adenoma* *7*	*True_Carcinoma* *19*	Accuracy (True positive rate) 73.07%
	False omission rate *0.29*	Positive predictive value *0.76*	*F*_0.5_-score: 0.7540
			*F*_1_-score: 0.7451
			*F*_2_-score: 0.7364
			*G*-mean: 0.7452

**Table 7:** Result of the CNN inference conducted on the test data groups, both H_A_ and H_B_.

	Predicted_Adenoma	Predicted_Carcinoma		Overall accuracy
H_A_	*True_Adenoma* *100%*	*False_Carcinoma* *0.00%*	True negative rate 1.0	*100%*
	*False_Adenoma* -	*True_Carcinoma* -	True positive rate -	
	False omission rate -	Positive predictive value -	*F*_0.5_-score: -	
			*F*_1_-score: -	
			*F*_2_-score: -	
			*G*-mean: -	

H_B_	*True_Adenoma* *69.76%*	*False_Carcinoma* *30.24%*	True negative rate 0.6976	*70.37%*
	*False_Adenoma* *28.95%*	*True_Carcinoma* *71.05%*	True positive rate 0.7105	
	False omission rate 0.2683	Positive predictive value 0.6749	*F*_0.5_-score: 0.6818	
			*F*_1_-score: 0.6923	
			*F*_2_-score: 0.7031	
			*G*-mean: 0.6925	

Total	*True_Adenoma* *93.19%*	*False_Carcinoma* *6.81%*	True negative rate 0.9319	*89.52%*
	*False_Adenoma* *28.95%*	*True_Carcinoma* *71.05%*	True positive rate 0.7105	
	False omission rate 0.0582	Positive predictive value 0.6750	*F*_0.5_-score: 0.6818	
			*F*_1_-score: 0.6923	
			*F*_2_-score: 0.7031	
			*G*-mean: 0.6925	

**Table 8:** Result of various typical inference model.

	Predicted_Adenoma	Predicted_Carcinoma		Overall Accuracy
SVM	*True_Adenoma* *18.30%*	*False_Carcinoma* *81.70%*	True negative rate 0.183	*40.96%*
	*False_Adenoma* *22.92%*	*True_Carcinoma* *77.08%*	True positive rate 0.7708	
	False omission rate 0.4400	Positive predictive value 0.3718	*F*_0.5_-score: 0.4148	
			*F*_1_-score: 0.5017	
			*F*_2_-score: 0.6346	
			*G*-mean: 0.5354	

KNN	*True_Adenoma* *91.50%*	*False_Carcinoma* *8.50%*	True negative rate 0.9150	*63.45%*
	*False_Adenoma* *81.25%*	*True_Carcinoma* *18.75%*	True positive rate 0.1875	
	False omission rate 0.3578	Positive predictive value 0.5806	*F*_0.5_-score: 0.4091	
			*F*_1_-score: 0.2835	
			*F*_2_-score: 0.2169	
			*G*-mean: 0.3299	

ANN	*True_Adenoma* *79.08%*	*False_Carcinoma* *20.92%*	True negative rate 0.7908	*70.28%*
	*False_Adenoma* *43.75%*	*True_Carcinoma* *56.25%*	True positive rate 0.5625	
	False omission rate 0.2577	Positive predictive value 0.6279	*F*_0.5_-score: 0.6136	
			*F*_1_-score: 0.5934	
			*F*_2_-score: 0.5745	
			*G*-mean: 0.5943	

Normal Bayes Classifier	*True_Adenoma* *38.56%*	*False_Carcinoma* *61.44%*	True negative rate 0.3856	*57.03%*
	*False_Adenoma* *13.54%*	*True_Carcinoma* *86.46%*	True positive rate 0.8646	
	False omission rate 0.1805	Positive predictive value 0.4689	*F*_0.5_-score: 0.5162	
			*F*_1_-score: 0.6081	
			*F*_2_-score: 0.7398	
			*G*-mean: 0.6367	

**Table 9:** Comparison result of diagnostic performance with other USFNA method [5] for follicular thyroid neoplasm.

(%)	FS (Frozen Section)	USFNA	Our proposed
Sensitivity	80.0 (24/30)	84.2 (48/57)	71.05 (27/38)
Specificity	96.3 (77/80)	52.2 (36/69)	93.19 (178/191)
PPV	88.9 (24/27)	59.3 (48/81)	67.49 (27/40)
NPV	92.8 (77/83)	80.0 (36/45)	89.89 (178/189)
Accuracy	91.8 (101/110)	66.7 (84/126)	89.52 (205/229)
